# Supplementary material for: Gate controlled anomalous phase shift in Al/InAs Josephson junctions
Source: Nat Commun. 2020 Jan 10;11:212. doi: 10.1038/s41467-019-14094-1 (PMC6954213; doi:10.1038/s41467-019-14094-1)
Supplement: Supplementary file 1 — Supplementary Information [file 41467_2019_14094_MOESM1_ESM.pdf]

Gate Controlled Anomalous Phase Shift in Al/InAs Josephson Junctions

Mayer et al.

Supplementary Information

Supplementary Discussion

The application of an in-plane magnetic field on the sample leads to a reduction of the critical current of the Josephson and a distortion of the Fraunhoffer pattern as illustrated in Supplementary Figure 1.

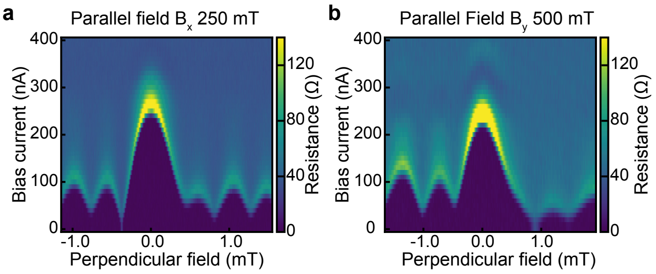

Supplementary Figure 1.(Color online) Fraunhofer pattern of JJ 1 in the presence of an in-plane field ($V_g^1 = 0V$, $V_g^2 = -7V$). a) Fraunhofer pattern when applying 250 mT along the x direction i.e. parallel to the current. b) Fraunhofer pattern when applying 500 mT along the y direction.

The change in the critical current of the junction appears to strongly depends on the direction of the applied in-plane field. In Supplementary Figure 1, the amplitude of the critical current is similar in both plots but the magnitude of the applied magnetic field is twice as large in the y direction compared to the x direction.

For both directions of the field, the Fraunhofer pattern appears asymmetric which is not the case in the absence of the in-plane as illustrated in the main text. The observed distortions are similar for both orientations of the field. Despite these distortions a clear central peak remains at all magnetic fields below B_c. Additionally, as stated in the main text, the period of Fraunhofer oscillations is unchanged. This indicates there are not large deviations from a uniform current distribution even in the presence of large in-plane magnetic fields.

When comparing those data to the ones presented in the main text, one can notice that the width of the first node has been divided by about two. We attribute this effect, which is also visible in the SQUID oscillations, to the transition out of the superconducting state of the indium layer at the back of the sample. The transition occurs around 30 mT and does not impact our study otherwise.

To alleviate any concern of the reader may have regarding the fact that we plot most of our data as a function of the phase of the SQUID, we plot in Supplementary Figure 2 the data of the middle panel of Fig. 3 as a function of the out-of-plane magnetic field. We would like however to underline here that when fitting our data a single frequency is used for all the data presented together and as a consequence the relationship between the SQUID phase and the magnetic field is linear. Furthermore since the data at different gates are acquired within a single magnetic field field there cannot be arbitrary phase offsets in the SQUID from one gate voltage to the next.

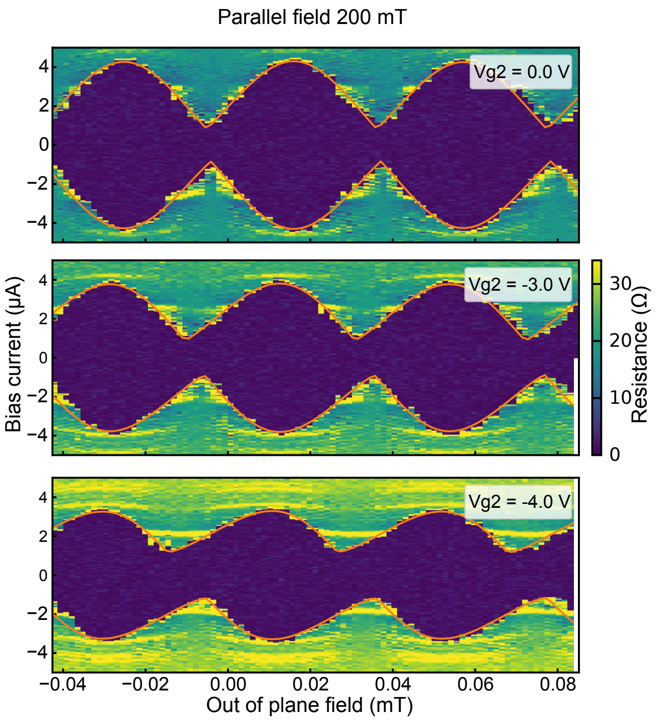

Supplementary Figure 2. (Color online) Resistance of the device as a function of the out-of-plane magnetic field and the bias current at 200 mT and three different values of V_g^2

The current phase relationship (CPR) of a Josephson junction with a high transparency present a notable sawtooth like profile which leads to distortions of the typical SQUID oscillations. In the following we discuss how this affects our measurements.

In Supplementary Figure 3, we present calculations performed for two junction of varying critical currents and transparencies. For junctions with different transparencies, it appears that changing the relative amplitude of the current in each arm, $a = \frac{I_1}{I_2}$ of the SQUID does not alter the position of the maximum of the oscillation even though it can strongly alter the shape of the oscillation. This should not be surprising since the phase difference to be at the maximum of both CPR only depends on the shape of the CPR. This validates our method of extraction of the phase shift under the assumption that the applied gate voltage does not affect the junction transparency.

In Supplementary Figure 4 we illustrate the artificial phase-shift that can be induced by varying the transparency of one junction while the other is kept at a fixed transparency (0.5). We consider equal current in each arm, but as mentioned above this has no consequence on the phase-shift. As the transparency is varied between 0 and 0.99, the oscillations are shifted by about 0.25π which is about half of the largest phase-shift we measured. Furthermore that shift has the opposite sign on the positive and negative branches of the SQUID critical current, which allows us to rule out this effects as being the dominant mechanism in our experiment as illustrated

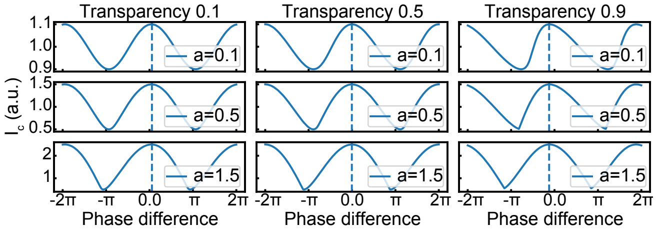

Supplementary Figure 3. (Color online) SQUID critical current for highly transparent junction. The critical current of one of the junction is fixed to 1 and its transparency is set to 0.5. The values used for the other junction are the ones indicated on the figure. The method of calculation of the plotted current is the same one used to fit the experimental data. The dashed lines indicate the position of the maximum of the oscillation.

in Fig. 3 of the main text.

To reduce the measurement time, we have often worked with only the positive branch of the SQUID critical current and assumed a constant transparency of the junction as a function of the gate. This can lead to errors in the determination of the phase-shift obviously but as discussed above we have checked that a varying transparency cannot alone explain all our results.

The application of a gate voltage on the junctions may alter the current distribution and hence the effective area of the SQUID. We examine here this possibility to ascertain it cannot explain our results.

Let's consider an initial situation with a out of plane field B applied to the SQUID of surface S such that the enclosed flux is $n\,\phi_0$, where ϕ_0 is the quantum of flux. When applying the gate let's assume that the surface enclosed becomes $S + \Delta S$, such that the flux becomes $(n + x)\phi_0$. From this simple argument we can conclude that $x/n = \Delta S/S$. If we consider the case of the largest phase-shift we observed $\sim \pi/2$, which corresponds to a quarter of flux and since we always work close to the maximum of the Fraunhofer pattern let's take $n = 5$. To explain our observation, the surface of the SQUID would have to change by 5% which given the the surface of our SQUID (25 μm^2) and the surface of our junctions ($100nm \times 1\mu m$) is not possible even taking into account flux focusing. Flux focusing increases the effective surface of the junction by concentrating the magnetic flux lines inside the junction. However based on the comparison of the expected Fraunhofer frequency to the measured one, its impact doubles at most the effective area of the junction.

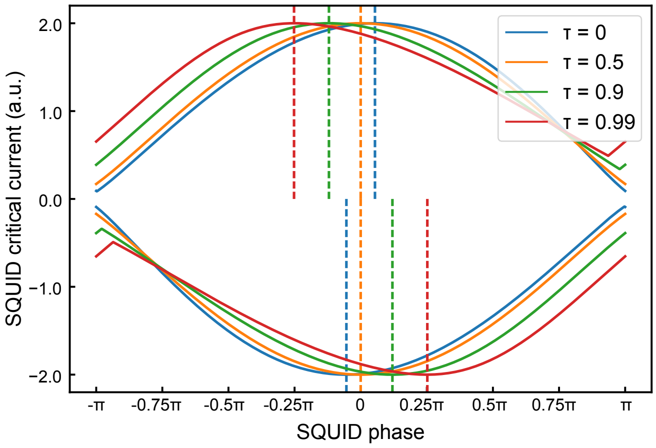

Supplementary Figure 4. (Color online) SQUID critical current (positive/negative) for varying transparency of one junction. The transparency of the other junction is fixed at 0.5 and the current in both amplitudes are taken equal. The dashed lines indicate the position of the maximum/minimum of the oscillation.

The phase-shift of JJ2 as a function of the applied field presented in Fig. 4 of the main text has been extracted by fitting the SQUID oscillations of both JJ1 and JJ2 in a constrained manner as described in the Methods section of the main text. We present in Supplementary Figure 5, the data and fits obtained at three different values of magnetic field. As in the main text, we mark the position of the maximum at Vg = -4 V using a dashed line and the position of the maximum at each field using a star.

One can observe that the phase-shift observed for JJ1 is of the same order of magnitude than the one for JJ2 but of the opposite sign as expected from the SQUID equation.

According to most theoretical predictions, in the absence of Dresselhaus spin-orbit coupling applying a magnetic field along the x axis should not give rise to an anomalous phase. In InAs, the spin-orbit interaction is expected to be mostly of the Rashba type and we hence expect a reduction of the phase shift by rotating the field.

We present in Supplementary Figure 6, data taken in the presence of a 300 mT field at 45 (a) and along the x-axis (b) along with the extracted phase-shift as the function of the angle θ defined in Figure 1 c of the main text.

The phase-shift appears to diminish as we rotate the field away from the y-axis but remains finite as illustrated in (a) and (b). The error bars on the determination of the phase-shift are large due to fluctuations of the SQUID period inside the dataset (up to maximum of 10%) that forced us to treat it in two separate subsets.

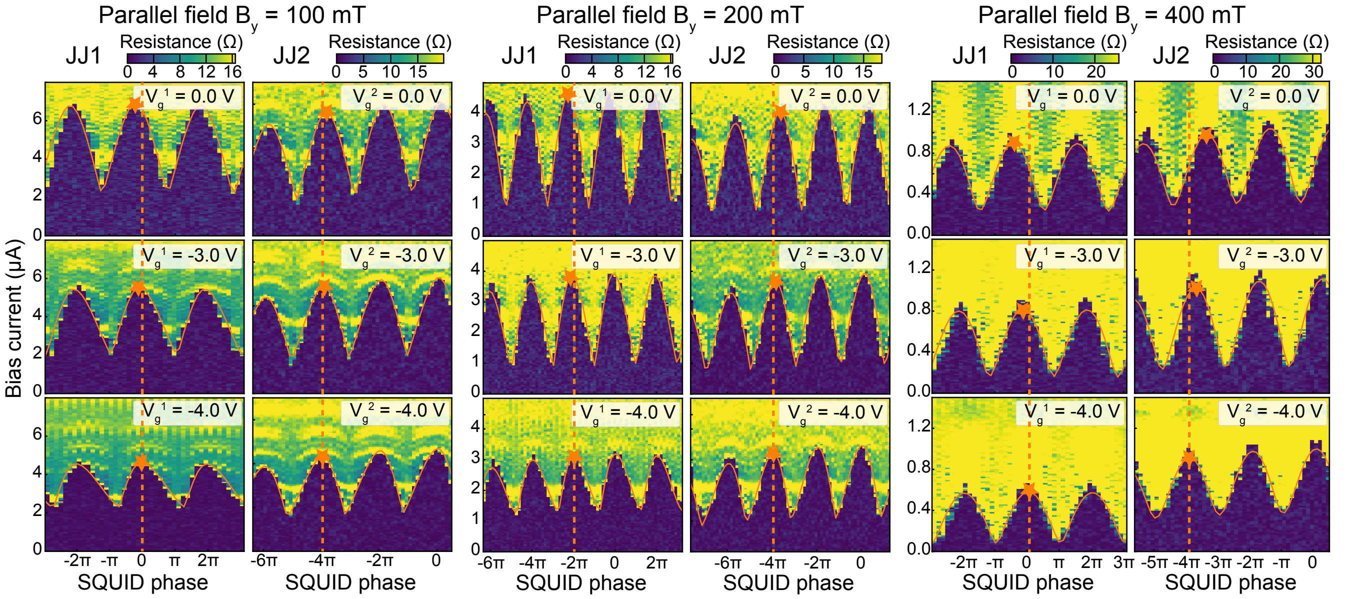

Supplementary Figure 5. (Color online) Fits performed simultaneously (see Methods) on JJ1 and JJ2 data to extract the phase shift. When working on JJ1, Vg2 is set to 0 V, when working on JJ2, Vg1 is set to -2 V

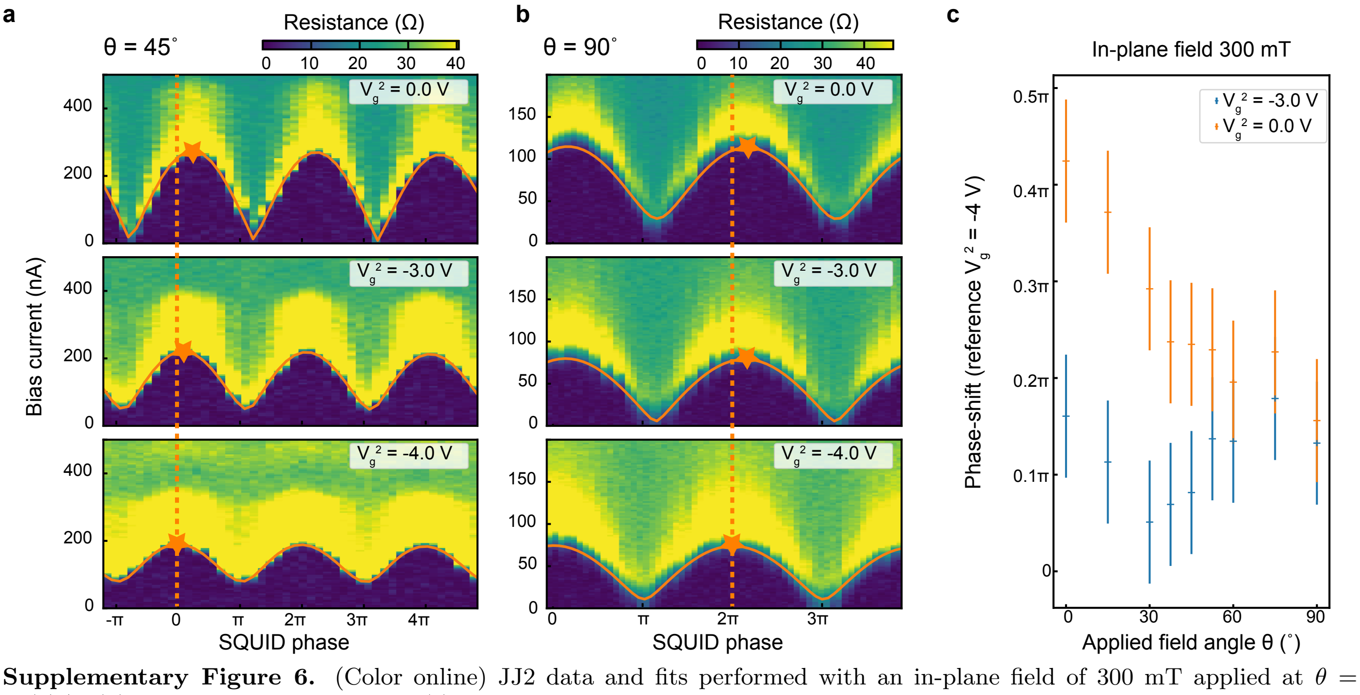

Supplementary Figure 6. (Color online) JJ2 data and fits performed with an in-plane field of 300 mT applied at θ = 45(a)/90(b) with respect to the y-axis. (c) Phase-shift extracted from the fits as a function of θ. Error bars indicate uncertainly due to fluctuations of SQUID period.
